# EndoFLIP Guided Assessment of Pyloric Distensibility Identifies Associations With Delayed Gastric Emptying and Symptoms of Gastroparesis

**DOI:** 10.1111/nmo.70405

**Published:** 2026-07-27

**Authors:** Subhankar Chakraborty, Adam Spandorfer, Flavio Bonilla, Kebire Gofar, Ladan Navari

**Affiliations:** ^1^ Division of Gastroenterology, Hepatology and Nutrition The Ohio State University Wexner Medical Center Columbus Ohio USA; ^2^ Department of Internal Medicine The Ohio State University Columbus Ohio USA; ^3^ University of Wisconsin‐Madison Madison Wisconsin USA

## Abstract

**Background:**

Pyloric dysfunction assessed by EndoFLIP has been implicated in the pathogenesis of gastroparesis. However, the relationship between pyloric dysfunction, gastric emptying, and severity of gastroparesis symptoms remains unclear.

**Aim:**

Investigate the association of pyloric distensibility index (DI) and area under the curve (AUC) for a DI to volume curve with percent of gastric retention and severity of gastroparesis symptoms.

**Methods:**

We retrospectively reviewed the electronic charts of 321 adult patients who underwent graded pyloric sphincter distension over a range of 30–70 mL balloon volume using an EF‐322 EndoFLIP catheter during a sedated upper endoscopy. To be included in the analysis, participants should have also completed questionnaires about severity of gastroparesis symptoms before EndoFLIP (*n* = 229). Gastric emptying study (GES) test results completed within the last 10 years were abstracted from chart review (*n* = 132). AUC for adjacent balloon volumes (40–50, 50–60 and 60–70 mL) were calculated using the trapezoid formula. Associations between DI, AUC, gastric emptying and GI symptoms were assessed using non‐parametric independent sample tests, and Spearman's correlation. Receiver operating characteristic (ROC) curve was used to calculate the ability of DI and AUC to distinguish between those with normal and abnormal gastric emptying.

**Results:**

Median age of the study population was 50 years (IQR 20.0). Majority of patients were female (79.9%), and White (89.1%). More than half of those with prior GES had gastroparesis (*N* = 68, 51.5%). Pyloric DIs at 60 and 70 mL and AUCs at 50–60 and 60–70 mL were smaller in those with delayed gastric emptying compared to those with normal gastric emptying. Pyloric DI at 50 mL (rho = −0.16, *p* = 0.014) and AUCs at 40–50 mL (rho −0.16, *p* = −0.018) and 50–60 mL (rho = −0.16, *p* = 0.019) showed significant associations with severity of stomach fullness, decreasing as severity of the symptom worsened. Pyloric DI of 8.9 mm^2^/mmHg at 60 mL (94.7% sensitive, 26.2% specific, AUC 0.61, *p* = 0.029) and 6.25 mm^2^/mmHg at 70 mL (92.5% sensitive, 31% specific, AUC 0.62, *p* = 0.038) distinguished between normal and delayed gastric emptying. In linear mixed‐effects modeling, anesthesia type demonstrated a volume‐dependent effect on pyloric DI, with monitored anesthesia care (MAC) associated with higher DI than general anesthesia (GA) at increasing balloon volumes (anesthesia × volume *p* < 0.001). Further, female sex was independently associated with lower DI (β −3.55, *p* = 0.002), but this was offset by a significant anesthesia × gender interaction, whereby females exhibited relatively higher DI under monitored anesthesia care compared to general anesthesia (β +3.49, *p* = 0.005).

**Conclusion:**

Pyloric sphincter DI and AUC at higher balloon volumes differentiated those with normal from those with delayed gastric emptying. The relationship with severity of gastroparesis symptoms was limited to stomach fullness. The effect of type of anesthesia on pyloric distensibility depends on balloon volume and sex of the patient. Pyloric DI and AUC at 60 and 70 mL could be helpful to phenotype the pyloric sphincter in patients with gastroparesis symptoms.

## Key Points

1. Pyloric distensibility measured at higher EndoFLIP balloon volumes (60–70 mL) is associated with delayed gastric emptying, whereas measurements at lower volumes show no significant association.
2. AUC metrics derived from the pyloric distensibility‐volume relationship showed significant associations with delayed gastric emptying, supporting dynamic assessment of pyloric function beyond single‐volume measurements.
3. Pyloric DI thresholds of ≤ 8.9 mm^2^/mmHg at 60 mL and ≤ 6.25 mm^2^/mmHg at 70 mL identified delayed gastric emptying with high sensitivity (> 89%).
4. Anesthesia demonstrated significant volume‐dependent and sex‐dependent effects on pyloric distensibility during EndoFLIP assessment.

## Graphical abstract

Measuring pyloric distensibility at high balloon volumes using EndoFLIP differentiates normal from delayed gastric emptying, while pyloric stiffness specifically correlates with increased subjective feeling of stomach fullness. EndoFLIP could be a valuable clinical tool to phenotype the pyloric sphincter in symptomatic patients.

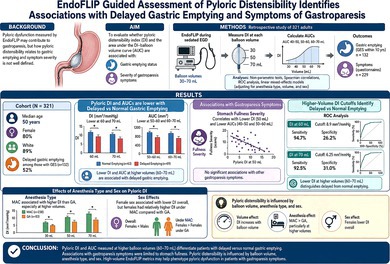

## 1 Background

Gastroparesis is a complex functional sensory‐motor disorder characterized by delayed gastric emptying in the absence of mechanical obstruction [1]. While the gastric emptying scan (GES) remains the diagnostic gold standard, a persistent clinico‐pathological paradox exists: the severity of gastric retention often correlates poorly with the intensity of patient symptoms [2]. Recent evidence suggests that the pathophysiology extends beyond simple antral pump failure, pointing toward the pylorus as a key determinant of gastric outflow [3, 4].

The pylorus acts as the gatekeeper of gastric emptying, regulating the flow of chyme into the duodenum through coordinated relaxation and contraction. Recent reviews have highlighted the importance of understanding the role of pyloric sphincter in the pathophysiology of symptoms of gastroparesis [3, 4]. The EndoFLIP test has emerged as a novel technique to assess the dimensions of the sphincter opening and its mechanistic properties. It allows us to measure metrics like diameter, intra‐balloon pressure, and from these two calculate the distensibility index (DI). The higher the DI, the more compliant the sphincter. There have been several articles that have described differences in pyloric sphincter distensibility using EndoFLIP between healthy individuals and those with gastroparesis [5, 6, 7]. These studies have usually used FLIP balloon volumes between 40 and 60 mL. One of these included 24 healthy volunteers who underwent unsedated upper endoscopy with EndoFLIP. They reported that the distensibility of the pyloric sphincter increased as balloon volume increased ranging from 13 to 17 mm^2^/mmHg [8]. Another one in 21 healthy unsedated patients described the pyloric distensibility to be 9.7 mm^2^/mmHg at 40 mL [5]. A third study reported that the median pyloric distensibility was 8.4 mm^2^/mmHg at 40 mL in 20 healthy individuals who underwent the procedure under propofol anesthesia [6]. These studies are limited by their small size. Unsedated endoscopy for EndoFLIP is difficult in clinical practice due to patient discomfort. Studies in patients with gastroparesis have examined the differences in distensibility compared to healthy individuals, differences between diabetic and idiopathic gastroparesis, and the effect of pyloric therapies like balloon dilation, POEM, and botulinum toxin injection on pyloric distensibility. One study of 46 patients with diabetic gastroparesis, 33 with idiopathic gastroparesis, and 21 healthy volunteers found that the average pyloric distensibility at 40 mL was lower in gastroparesis patients compared to healthy individuals (between 10.8 and 14.8 mm^2^/mmHg in gastroparesis compared with 25.2 mm^2^/mmHg in healthy volunteers). No correlation was found between pyloric sphincter measurements and diabetes‐related biochemical characteristics, such as blood glucose, hemoglobin A1c, type of diabetes mellitus, presence of neuropathy, and use of GLP1 agonists [7]. Another study in 54 patients with gastroparesis found that the pyloric sphincter contour was best seen at 40 mL balloon volume. This study noted that the range of pyloric sphincter distensibility was very wide (1–55 m^2^/mmHg) and that there was no correlation between pyloric DI at 40 mL and symptoms of gastroparesis [9]. There was no correlation between the percentage of test meal retention in the stomach after 4 h and pyloric cross‐sectional area, pressure, or distensibility in 44 patients with gastroparesis (30 idiopathic and 14 diabetics). The study used an EF‐325 EndoFLIP catheter and measurements were taken at balloon volumes of 30 and 40 mL. There was also no significant association between pyloric distensibility and the severity of nausea, retching, vomiting, early satiety, post prandial fullness, loss of appetite, or upper abdominal pain [10].

Pyloric distensibility at baseline has been reported to be associated with improvement in gastroparesis symptoms in patients undergoing pyloric therapy. In one study of 13 patients who underwent through the scope pyloric dilation due to persistent symptoms after pyloroplasty or pyloromyotomy, pyloric DI at 30, 40, and 50 mL were significantly smaller in those patients who did not improve after balloon dilation (4.7 vs. 13.2 mm^2^/mmHg, 7.2 vs. 13.9 mm^2^/mmHg, and 4.9 vs. 10.2 mm^2^/mmHg respectively) [11]. A similar finding was noted in a study of 35 patients with gastroparesis symptoms refractory to therapy who underwent EndoFLIP before pyloric botulinum toxin injection. Stomach fullness and bloating were differentially improved after botulinum toxin injection only in those patients who had normal (defined as ≥ 10 mm^2^/mmHg) pyloric distensibility at 40 mL volume before botulinum toxin injection [12].

A study of 184 patients using the EF‐322 catheter found that DI measurement at 65 mL intraballoon volume was able to distinguish between patients with wide versus tight pyloric sphincter on visual inspection on endoscopy [13]. A strong resistance to pyloric intubation predicted abnormal pyloric DI (< 8 mm^2^/mmHg) in only 59% of patients suggesting that visual inspection alone was not adequate at assessing pyloric dysfunction.

A recent review by Varghese et al. reviewed the role of EndoFLIP in assessing response to gastric per oral endoscopic myotomy (G‐POEM) [14]. Five studies, with sample sizes between 13 and 34, showed variable results as to whether pyloric DI predicted response to G‐POEM.

From the aforementioned studies, it is evident that most studies on pyloric distensibility using EndoFLIP have been small. Further, all the studies have investigated distensibility measurements at discrete volumes of FLIP balloon distension. Given that pyloric distension is a dynamic process, measurement at a single point can only provide partial information about pyloric function. A more comprehensive assessment would incorporate behavior of the sphincter over a range of volumes.

Given the lack of large studies investigating the relationship between pyloric distensibility, percentage of gastric retention, and severity of gastroparesis symptoms, we conducted a retrospective study investigating this question. We examined the association of pyloric DI at discrete balloon volumes and area under the DI‐volume curve with the percent of gastric retention after 4 h and the severity of gastrointestinal symptoms.

## 2 Methods

### 2.1 Study Design and Participants

**FIGURE 1:**
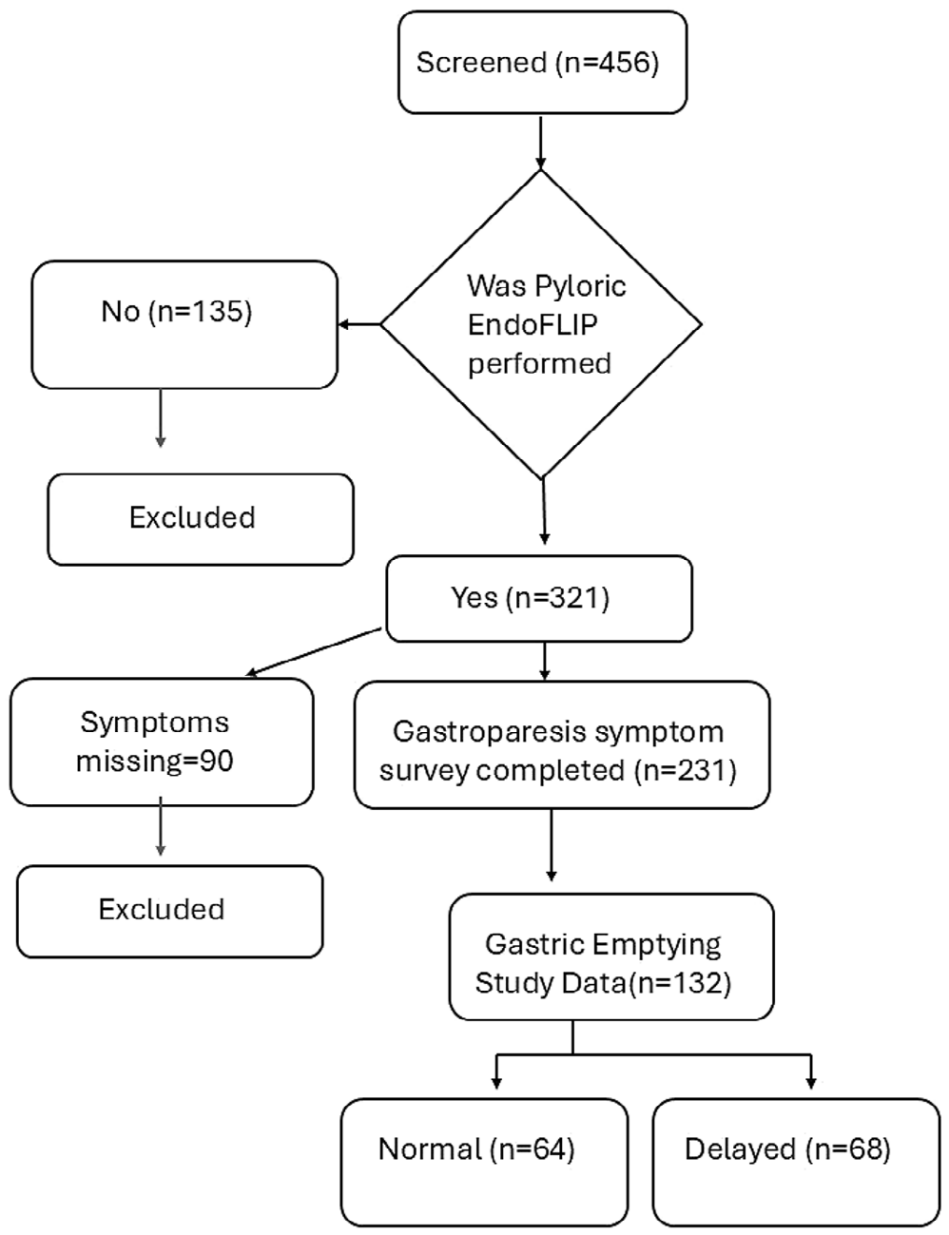
Schematic showing the inclusion and exclusion criteria for the study. All patients undergoing EndoFLIP procedure were older than 18 years at the time of their procedure.

We performed a retrospective cohort analysis of adult patients who underwent pyloric functional luminal imaging probe (EndoFLIP) assessment due to various upper abdominal and esophageal symptoms (including gastroparesis, functional dyspepsia, chronic nausea, dysphagia, eosinophilic esophagitis, GERD, and achalasia). We pooled data from two EndoFLIP databases under separately approved IRB protocols. For the retrospective EndoFLIP registry (Study number 2022H0145), consent was waived. For the prospective EndoFLIP registry (Study number 2022H0130), participants completed the consent form either on paper or electronically using RedCap. To be eligible for inclusion in the analysis, the patient must have undergone pyloric EndoFLIP and also completed a questionnaire that enquired about the severity of upper GI symptoms before their EndoFLIP. A subset of patients had a gastric emptying study (GES) performed within 10 years from the time of the EndoFLIP. This time frame for GES is based on previously published clinical trial in gastroparesis [15]. Figure 1 shows how participants were selected for analysis. Demographics (age, sex, race) were abstracted from the electronic medical record.

### 2.2 EndoFLIP Measurements

EndoFLIP procedures were performed using an EF‐322 catheter (Medtronic, Minneapolis, MN, USA) as part of a standardized in‐house protocol for diameter and distensibility index measurement. While the EF‐325 catheter has generally been reported for assessment of pyloric sphincter distensibility, a large study of 184 patients reported using the EF‐322 catheter for pyloric sphincter assessment specifically because it provides a greater range of balloon volume measurements than the EF‐325 catheter and also allows for assessment of esophageal motor function during the same endoscopy procedure [13]. As most patients referred for EndoFLIP report both gastric and esophageal symptoms, we consistently use the EF‐322 catheter for our EndoFLIP procedures. Upper endoscopy procedures were performed between January 1, 2020 and January 20, 2025. Indications for EndoFLIP procedure included investigation of persistent and unexplained symptoms of the upper gastrointestinal tract localized to the esophagus (e.g., achalasia, dysphagia, gastroesophageal reflux disease) or stomach (gastroparesis, functional dyspepsia, chronic nausea). The results of pyloric EndoFLIP were used to guide decision making, specifically pylorus directed therapy such as balloon dilation (up to 20 or 30 mm using EsoFLIP), botulinum toxin injection or referral for POEM or pyloroplasty. The EndoFLIP catheter was positioned across the pylorus under endoscopic guidance, and measurements were obtained at prespecified distension volumes. Measurements were obtained at 10 cc increments from 30 to 70 mL. The EndoFLIP catheter was positioned by grasping its tip with a 15 mm snare outside the patient and then guiding the catheter using a standard upper endoscope into the 2nd portion of the duodenum. The snare was opened, the FLIP catheter tip released and the snare along with the scope then withdrawn into the stomach, leaving the catheter in the duodenum. If the catheter slipped back into the stomach, it was gently guided into the duodenum by applying pressure with the endoscope, a method we call the “sliding method”. While previous studies have described using a therapeutic upper endoscope, this kind of scope was not routinely available at our center and so we developed this technique for positioning the EndoFLIP catheter. The EndoFLIP measurements were performed after completion of a standard endoscopic examination, under direct vision with the tip of scope positioned at a distance from the balloon so as to not touch the balloon but be able to visualize the position of the balloon across the pylorus. For each distension volume, we recorded the maximum values for distensibility (DI). We waited at least 15 s after each target volume was reached to allow the measurements to stabilize. All EndoFLIP procedures were done by a single operator (SC). For our analysis, we used FLIP balloon volumes between 40 and 70 mL as we observed that at 30 mL the FLIP balloon did not have enough volume to come in contact with the margins of the pyloric sphincter. Most of the upper endoscopies were conducted under general anesthesia as per institutional protocol to reduce the risk of aspiration. The medications used during anesthesia included 2% lidocaine and propofol. If general anesthesia was used, then succinylcholine or rocuronium were used for induction only. Other medications used at the anesthetist's discretion included ondansetron, dexamethasone, midazolam and fentanyl.

### 2.3 Gastric Emptying Scintigraphy and Outcome Definition

Gastric emptying was assessed by scintigraphy. These were either conducted at our institution or an outside institution when clinically indicated. We categorized gastric emptying as normal, delayed, or rapid based on the percentage of retention at 1 h (< 30% indicates rapid emptying) and 4 h (> 10% indicates delayed emptying) or the time taken for half of the test meal to be emptied from the stomach. To test the association between DI or AUC and gastric retention, we tested three different cut‐offs for abnormal gastric retention at 4 h: > 10%, > 20%, and > 30%. Due to the small number of patients, we excluded those who had rapid (*n* = 4) or indeterminate gastric emptying (*n* = 1).

### 2.4 Calculating Area Under the Curve

Distensibility measurements performed at a given volume may miss the effect of change in volume on the overall biomechanical behavior of the pyloric sphincter. In order to capture overall pyloric mechanical behavior across the range of distension volumes, we calculated the area under a scatter plot curve (AUC) with the DI on the *y*‐axis and the balloon volume on the *x*‐axis. We used the trapezoidal method, a widely used method in pharmacokinetics and to study lower esophageal sphincter function distensibility, to calculate the AUCs between adjacent volumes [16, 17]. AUC was computed as the average DI between adjacent volumes multiplied by the change in balloon volume: [(DI_vol1_ + DI_vol2_)/2 × (Vol2‐Vol1)]. AUCs were calculated across four predefined distension ranges: 40–50 mL, 50–60 mL, and 60–70 mL.

### 2.5 Gastroparesis Symptoms

Participants completed a questionnaire where they rated the severity of nausea, retching, vomiting, stomach fullness, inability to finish a meal, post prandial fullness, bloating, and abdominal distension in the preceding 24 h on a scale ranging from 0 (None) to 4 (Very Severe). The questionnaire was completed either before their EndoFLIP procedure or during a clinic visit preceding the procedure. Although the symptom questionnaire was adapted from the validated Gastroparesis Cardinal Symptom Index [18], we used a shortened recall period as they were used as a practical instrument to ascertain clinical symptoms before the EndoFLIP procedure.

### 2.6 Statistical Analysis

To investigate the associations between pyloric distensibility and gastroparesis symptom severity and pyloric distensibility, we compared pyloric DI's and AUCs at five degrees of severity for each symptom. Due to the non‐normal distribution of most of the DI values, we used the non‐parametric independent samples test with post hoc assessment. When overall group differences were statistically significant, post hoc pairwise comparisons were performed using Bonferroni correction for multiple comparisons. Median DI and AUC values are reported with inter quartile ranges. The *p*‐values reported for all non‐parametric tests are the adjusted *p*‐values. A *p*‐value < 0.05 was considered statistically significant and that between 0.05 and 1.0 marginally significant.

To investigate the effect of anesthesia on pyloric distensibility index (DI), a linear mixed‐effects model was used to account for within‐subject correlation arising from repeated measurements and to evaluate volume‐dependent pyloric physiology. DI was specified as the dependent variable. Fixed effects included anesthesia type (monitored anesthesia care vs. general anesthesia), gender, and balloon distension volume, along with prespecified interaction terms for anesthesia × gender and anesthesia × volume to assess effect modification by sex and to evaluate whether anesthetic effects varied across increasing levels of pyloric distension. Models were additionally adjusted for age and body mass index (BMI) as covariates. Race was initially included but excluded from the final model due to lack of association with the outcome and to improve model parsimony. A subject‐specific random intercept was included to account for repeated measurements within individuals. A compound symmetry covariance structure was specified for repeated measures across balloon distension volumes. Models were estimated using restricted maximum likelihood (REML), and degrees of freedom were calculated using the Satterthwaite approximation. Type III tests of fixed effects were used for hypothesis testing. Model fit was assessed using Akaike's Information Criterion (AIC) and Bayesian Information Criterion (BIC). Monitored anesthesia, male gender, and distension volume of 70 mL were taken as the reference categories. Statistical significance was defined as a two‐sided *p* value < 0.05. All statistical analyses were performed using IBM SPSS Statistics (version 29.0, IBM Corp., Armonk, NY).

## 3 Results

We screened 456 patients who had EndoFLIP procedure during upper endoscopy. Of these, 321 (70.4%) patients had pyloric EndoFLIP performed. Most of these (*n* = 265, 82.6%) were performed under general anesthesia. 229 out of the 321 (71.3%) had also completed questionnaires about gastroparesis symptoms and were included in the analysis. A subset (*n* = 180) of the 321 patients had a gastric emptying test done. Of these 180, 132 (73.3%) had also completed gastroparesis symptom questionnaires (Figure 1). Most (50.4%) of the GES were completed less than 1 year from the date of the EndoFLIP, with a smaller number completed between 1 and 2 years (12.2%), 2–3 years (16.5%), and more than 3 years (20.9%) from the date of EndoFLIP. The demographics of the patients who had pyloric EndoFLIP subdivided by whether they completed gastroparesis questionnaires is summarized in Table S1.

### 3.1 Association Between Pyloric EndoFLIP Measurements and Gastric Emptying

#### 3.1.1 Distensibility Index

We first compared the pyloric distensibility between patients with normal or delayed gastric emptying. Delayed emptying was defined as retention more than 10%, 20% or 30% as defined in the methods section. The results are summarized in Table 1. Pyloric DI at 60 mL was significantly and at 70 mL marginally significantly smaller in patients with more than 10% retention after 4 h compared to those with lesser retention (*p* = 0.017 for 60 mL and 0.059 for 70 mL). DI at 60 and 70 mL were both lower in those with > 20% retention than in those with lesser gastric retention after 4 h (*p* = 0.03 for 60 mL and 0.048 for 70 mL). A similar pattern was observed when comparing those with more than 30% gastric retention at 4 h, DI at 60 and 70 mL was lower in those with more severe delays (*p* = 0.027 for 60 mL and *p* = 0.016 for 70 mL).

**TABLE 1:** Non‐parametric test comparing median DI between patients stratified by percent gastric retention and symptom severity.

	*N*	DI 40 mL		DI 50 mL		DI 60 mL		DI 70 mL
Gastric retention after 4 h								
> 10%	42	6.2 (5.1)		6.5 (4.5)		5.9 (3.8)		4.1 (2.2)
< 10%	50	6.6 (6.9)		6.8 (5.1)		6.4 (4.6)		4.7 (3.6)
	*p*	0.07		0.056		**0.017**		**0.059**
> 20%	38	5.5 (5.2)		5.9 (4.4)		5.8 (3.4)		4.1 (2.0)
< 20%	54	7.3 (6.3)		6.9 (4.9)		6.4 (4.6)		4.6 (3.3)
	*p*	0.07		0.11		**0.03**		**0.048**
> 30%	29	5.3 (5.1)		5.2 (3.5)		5.3 (3.0)		3.9 (1.7)
< 30%	63	7.5 (6.3)		7.2 (5.2)		6.4 (4.6)		4.6 (3.0)
	*p*	0.11		0.10		**0.027**		**0.016**
GI symptoms								
Nausea								
None	50	5.3 (5.1)	50	6.2 (3.6)	49	5.7 (4.0)	32	4.2 (2.9)
Mild	59	5.2 (4.4)	59	6.6 (6.0)	56	6.2 (5.1)	30	4.2 (3.2)
Moderate	51	5.2 (5.0)	50	6.4 (6.1)	49	5.0 (5.1)	37	3.8 (2.5)
Severe	37	6.0 (6.1)	37	6.4 (4.4)	37	5.9 (3.6)	28	4.5 (3.3)
Very severe	32	6.0 (5.2)	32	6.1 (5.8)	29	6.0 (4.6)	19	4.8 (2.2)
	*p*	0.87		0.96		0.99		0.52
Retching								
None	116	5.0 (4.9)	117	6.3 (5.9)	115	6.8 (5.0)	71	4.2 (2.7)
Mild	44	5.7 (5.2)	45	6.4 (4.7)	42	5.4 (4.2)	30	4.0 (2.1)
Moderate	27	8.0 (5.9)	29	6.6 (4.2)	29	6.3 (4.1)	23	4.7 (2.8)
Severe	26	5.5 (4.7)	26	6.0 (5.7)	24	6.8 (5.0)	19	4.6 (3.4)
Very severe	13	4.4 (8.4)	13	5.9 (8.1)	12	5.8 (4.5)	5	4.6 (2.1)
	*p*	0.28		0.70		0.85		0.82
Vomiting								
None	127	5.1 (4.7)	129	5.9 (5.5)	124	5.5 (3.7)	79	4.1 (2.7)
Mild	38	5.4 (5.6)	38	6.8 (5.9)	38	5.9 (3.9)	27	4.1 (2.1)
Moderate	21	5.3 (6.0)	22	5.7 (5.8)	22	5.6 (4.3)	15	4.7 (2.6)
Severe	19	5.1 (4.8)	20	5.5 (4.0)	19	4.6 (4.0)	13	4.0 (2.8)
Very severe	17	8.2 (7.1)	17	7.5 (5.7)	15	7.4 (3.7)	11	5.0 (3.1)
	*p*	0.33		0.45		0.52		0.69
Stomach fullness								
None	35	7.1 (5.7)	36	8.4 (5.3)	35	6.9 (3.4)	25	4.6 (2.5)
Mild	47	4.8 (5.6)	47	5.5 (6.3)	44	5.5 (4.0)	23	4.8 (3.1)
Moderate	65	5.3 (4.8)	66	5.9 (5.1)	63	4.9 (4.0)	43	3.7 (2.3)
Severe	41	4.7 (4.9)	42	6.4 (4.6)	41	5.6 (3.1)	30	4.2 (3.1)
Very severe	38	4.8 (5.0)	39	5.6 (4.7)	39	5.5 (4.2)	27	4.5 (2.1)
	*p*	0.11		**0.031**		**0.059**		0.68
Inability to finish a meal								
None	40	5.3 (5.0)	41	6.7 (6.7)	40	5.7 (4.0)	27	4.2 (2.0)
Mild	53	4.8 (4.7)	53	5.3 (3.3)	50	6.4 (4.0)	34	5.1 (3.0)
Moderate	58	5.3 (5.3)	59	6.2 (5.8)	58	5.5 (4.1)	33	3.8 (2.2)
Severe	36	6.4 (7.0)	37	6.4 (5.6)	35	5.0 (4.0)	29	4.1 (2.1)
Very severe	38	5.9 (5.1)	39	6.6 (5.2)	38	6.0 (4.3)		
	*p*	0.98		0.99		0.99		**0.028**
Excessive post prandial fullness								
None	34	6.1 (5.0)	35	7.2 (5.0)	33	6.2 (5.0)	22	4.3 (2.4)
Mild	47	4.8 (4.9)	47	5.3 (6.2)	46	5.2 (3.9)	28	4.3 (3.1)
Moderate	56	4.5 (5.7)	56	5.6 (5.9)	53	5.7 (4.0)	33	4.2 (1.9)
Severe	62	6.4 (4.8)	65	6.4 (5.0)	64	5.1 (4.0)	48	4.0 (3.3)
Very severe	23	5.1 (5.2)	23	5.6 (4.3)	22	4.9 (4.1)	13	4.4 (2.8)
	*p*	0.49		0.40		0.76		0.86
Loss of appetite								
None	66	6.2 (4.8)	68	6.9 (6.8)	64	5.9 (4.4)	38	4.5 (2.8)
Mild	56	4.8 (4.9)	56	6.8 (6.2)	55	6.3 (4.1)	38	4.2 (3.1)
Moderate	46	4.9 (5.4)	47	5.6 (3.9)	47	5.5 (3.9)	32	4.2 (2.1)
Severe	37	4.3 (4.6)	37	6.4 (3.9)	36	5.0 (3.7)	26	4.2 (3.3)
Very severe	20	6.1 (4.0)	21	5.9 (4.9)	19	6.0 (3.2)	13	4.6 (2.5)
	*p*	**0.076**		0.52		0.66		0.92
Bloating								
None	41	4.7 (5.2)	42	6.0 (5.5)	41	5.7 (4.4)	27	4.2 (2.7)
Mild	47	6.2 (5.4)	47	7.5 (6.6)	44	6.4 (4.3)	31	4.6 (2.9)
Moderate	42	5.4 (4.3)	42	5.9 (5.2)	40	5.5 (4.0)	27	4.2 (2.1)
Severe	44	4.8 (6.9)	45	6.3 (5.7)	44	5.0 (4.0)	30	3.8 (3.3)
Very severe	49	5.1 (4.7)	51	5.9 (4.4)	50	5.6 (3.0)	32	4.3 (1.9)
	*p*	0.37		0.24		0.66		0.98
Abdominal distension								
None	59	5.7 (4.0)	60	6.8 (5.1)	58	6.4 (4.2)	43	4.6 (2.7)
Mild	47	5.5 (6.0)	47	6.0 (5.7)	44	5.4 (4.4)	26	4.1 (3.1)
Moderate	40	4.8 (5.1)	40	5.9 (4.7)	38	5.1 (4.0)	24	4.3 (3.2)
Severe	31	4.3 (5.3)	32	6.3 (3.1)	32	5.2 (3.0)	22	3.9 (1.9)
Very severe	49	5.7 (5.0)	51	5.9 (5.4)	50	5.8 (4.4)	33	4.2 (2.3)
	*p*	0.69		0.68		0.81		0.88

*Note:* Table 1 summarizes the results of non‐parametric comparisons of median pyloric distensibility index (DI) measured at balloon volumes of 40, 50, 60, and 70 mL across increasing severities of gastroparesis symptoms and across thresholds of delayed gastric emptying. *p*‐values represent overall group differences across symptom severity categories (none to very severe) assessed using non‐parametric testing. Values were bolded to highlight those that were statistically significant.

There was a weak inverse correlation between pyloric DI at 60 and 70 mL and the percentage of gastric retention at 4 h (*r* = −0.239, *p* = 0.005 and *ρ* = −0.260, *p* = 0.011 respectively, Table S2) but not at 1 or 2 h. There was also a marginally significant inverse correlation between gastric retention at 4 h and pyloric DI and 40 and 50 mL (*ρ* = −0.147 and −0.139, *p* = 0.078 and 0.093). No associations were observed between DI and other balloon volumes.

#### 3.1.2 Area Under the Distensibility‐Volume Curve (AUC)

Next, we compared AUCs between those with normal and abnormal gastric retention. When compared with the AUC in patients whose gastric retention was less than the pre‐specified cut‐off, those with retention above the cut‐offs had significantly lower AUC, especially at higher balloon volumes. For instance, patients in whom the percentage gastric retention at 4 h was greater than 10%, 20%, or 30% had significantly smaller AUC_50–60mL_ (*p* = 0.019, 0.044, and 0.035, respectively), and AUC_60–70mL_ (*p* = 0.058, 0.039 and 0.025, respectively) than those whose gastric retention was less than the corresponding cut‐off value. However, no significant difference was seen between those with normal and abnormal gastric retention when compared by their AUC_40–50mL_ (Table 2).

**TABLE 2:** Nonparametric tests comparing AUCs with percent gastric retention and severity of gastroparesis symptoms.

	*N*	AUC 40–50 mL		AUC 50–60 mL		AUC 60–70 mL
Gastric retention after 4 h						
> 10%	50	58.0 (50.1)		59.3 (37.0)		49.8 (28.8)
< 10%	42	69.0 (48.0)		70.8 (37.0)		53.8 (39.6)
	*p*	0.075		**0.019**		**0.058**
> 20%	54	57.0 (46.9)		55.0 (37.6)		47.5 (28.3)
< 20%	38	69.8 (49.0)		70.8 (39.6)		53.0 (39.1)
	*p*	0.12		**0.044**		**0.039**
> 30%	63	56.5 (42.0)		49.0 (32.5)		46.5 (26.3)
< 30%	29	70.0 (50.0)		73.5 (47.0)		54.5 (35.5)
	*p*	0.12		**0.035**		**0.025**
Nausea						
None	50	59.5 (41.4)	49	58.0 (39.5)	32	48.0 (39.1)
Mild	57	55.5 (54.0)	56	61.3 (49.0)	30	53.0 (34.4)
Moderate	50	57.3 (50.3)	49	57.0 (56.3)	37	44.0 (34.8)
Severe	35	62.5 (47.5)	37	56.5 (36.8)	28	53.8 (33.4)
Very severe	32	57.3 (56.1)	29	60.5 (51.5)	19	61.0 (30.0)
	*p*	0.96		0.99		0.63
Retching						
None	116	65.1 (36.9)	115	66.8 (35.3)	71	54.1 (24.8)
Mild	44	64.4 (33.7)	42	64.8 (31.8)	30	51.2 (21.3)
Moderate	27	81.3 (50.9)	29	72.6 (37.7)	23	60.6 (23.7)
Severe	26	58.8 (31.9)	24	62.3 (32.5)	19	56.6 (28.3)
Very severe	13	72.3 (54.7)	12	56.7 (32.5)	5	50.9 (18.2)
		0.25		0.68		0.69
Vomiting						
None	127	57.5 (47.0)	124	57.5 (44.8)	79	46.5 (33.0)
Mild	38	59.5 (47.6)	38	65.3 (43.4)	27	51.5 (31.0)
Moderate	21	54.5 (58.0)	22	54.0 (53.5)	15	55.0 (35.5)
Severe	19	56.5 (38.0)	19	55.0 (32.5)	13	43.5 (33.8)
Very severe	17	78.5 (57.5)	15	80.0 (38.0)	11	67.5 (41.0)
	*p*	0.42		0.56		0.43
Stomach fullness						
None	35	75.5 (58.0)	35	78.5 (45.0)	25	52.0 (28.3)
Mild	47	54.0 (51.5)	44	59.8 (51.4)	23	51.5 (38.5)
Moderate	65	56.5 (48.5)	63	56.5 (43.5)	43	43.5 (34.5)
Severe	41	59.5 (44.5)	41	56.5 (34.5)	30	46.8 (34.1)
Very severe	38	52.0 (48.3)	39	55.5 (46.5)	27	50.5 (34.5)
	*p*	**0.025**		**0.023**		0.76
Inability to finish a meal						
None	40	63.0 (47.1)	40	60.0 (47.4)	27	51.0 (32.0)
Mild	53	54.0 (42.0)	50	61.0 (37.6)	34	57.8 (36.9)
Moderate	58	56.5 (60.0)	58	56.8 (54.6)	33	43.5 (33.0)
Severe	36	63.8 (60.4)	35	58.0 (40.0)	24	49.3 (30.3)
Very severe	38	59.0 (51.3)	38	61.0 (46.0)	29	50.5 (31.8)
	*p*	0.99		0.99		0.50
Excessive post prandial fullness						
None	34	68.8 (41.6)	33	68.0 (53.0)	22	50.3 (34.4)
Mild	47	54.0 (48.0)	46	61.3 (50.6)	28	42.8 (31.8)
Moderate	56	54.0 (65.1)	53	56.0 (42.0)	33	51.5 (36.5)
Severe	62	63.0 (47.6)	64	56.8 (41.5)	48	48.8 (37.4)
Very severe	23	54.5 (42.5)	22	55.3 (40.5)	13	46.5 (35.8)
	*p*	0.33		0.60		0.98
Loss of appetite						
None	66	68.8 (53.8)	64	61.5 (56.0)	38	48.0 (41.8)
Mild	56	57.8 (53.3)	55	63.0 (45.5)	38	53.0 (28.9)
Moderate	46	50.0 (44.0)	47	54.5 (36.0)	32	48.5 (34.9)
Severe	37	54.5 (37.8)	36	56.5 (37.3)	26	48.8 (35.1)
Very severe	20	57.3 (44.5)	19	64.5 (40.0)	13	53.0 (33.0)
	*p*	0.18		0.59		0.99
Bloating						
None	50	59.5 (41.4)	49	58.0 (39.5)	32	48.0 (39.1)
Mild	57	55.5 (54.0)	56	61.3 (49.0)	30	53.0 (34.4)
Moderate	50	57.3 (50.3)	49	57.0 (56.3)	37	44.0 (34.8)
Severe	35	62.5 (47.5)	37	56.5 (36.8)	28	53.8 (33.4)
Very severe	32	57.3 (56.1)	29	60.5 (51.5)	19	61.0 (30.0)
	*p*	0.29		0.41		0.99
Abdominal distension						
None	59	66.5 (42.0)	58	63.5 (41.4)	43	51.0 (32.5)
Mild	47	57.5 (57.5)	44	60.3 (55.3)	26	48.5 (37.6)
Moderate	40	51.8 (49.1)	38	55.3 (36.8)	24	54.8 (31.3)
Severe	31	55.0 (47.5)	32	56.8 (27.0)	22	48.0 (26.9)
Very severe	49	56.5 (52.5)	50	57.0 (47.6)	33	46.5 (36.3)
	*p*	0.57		0.72		0.84

*Note:* The bold text higlights that those *p* values were significant (*p* < 0.05).

Pyloric AUCs at higher balloon volumes (AUC_50–60mL_ and AUC_60–70mL_) demonstrated a weak inverse correlation with gastric retention at 4 h (*ρ* = −0.206, *p* = 0.015 and *ρ* = −0.264, *p* = 0.010, respectively) but not at 1 h or 2 h. AUCs at smaller balloon volumes (30–40 mL and 40–50 mL) did not show any correlation with percent gastric retention (Table S3).

### 3.2 Association Between Pyloric EndoFLIP Measurements and Gastroparesis Symptoms

#### 3.2.1 Distensibility Index

Next, we examined pyloric DI across varying severities of gastroparesis symptoms compared with the DI in those who reported absence of that specific symptom. Significant differences in DI were only observed for certain symptoms, namely stomach fullness, loss of appetite, and inability to finish a meal. Pairwise comparisons highlighted differences primarily between patients with no symptoms and those with higher severity.

**FIGURE 2:**
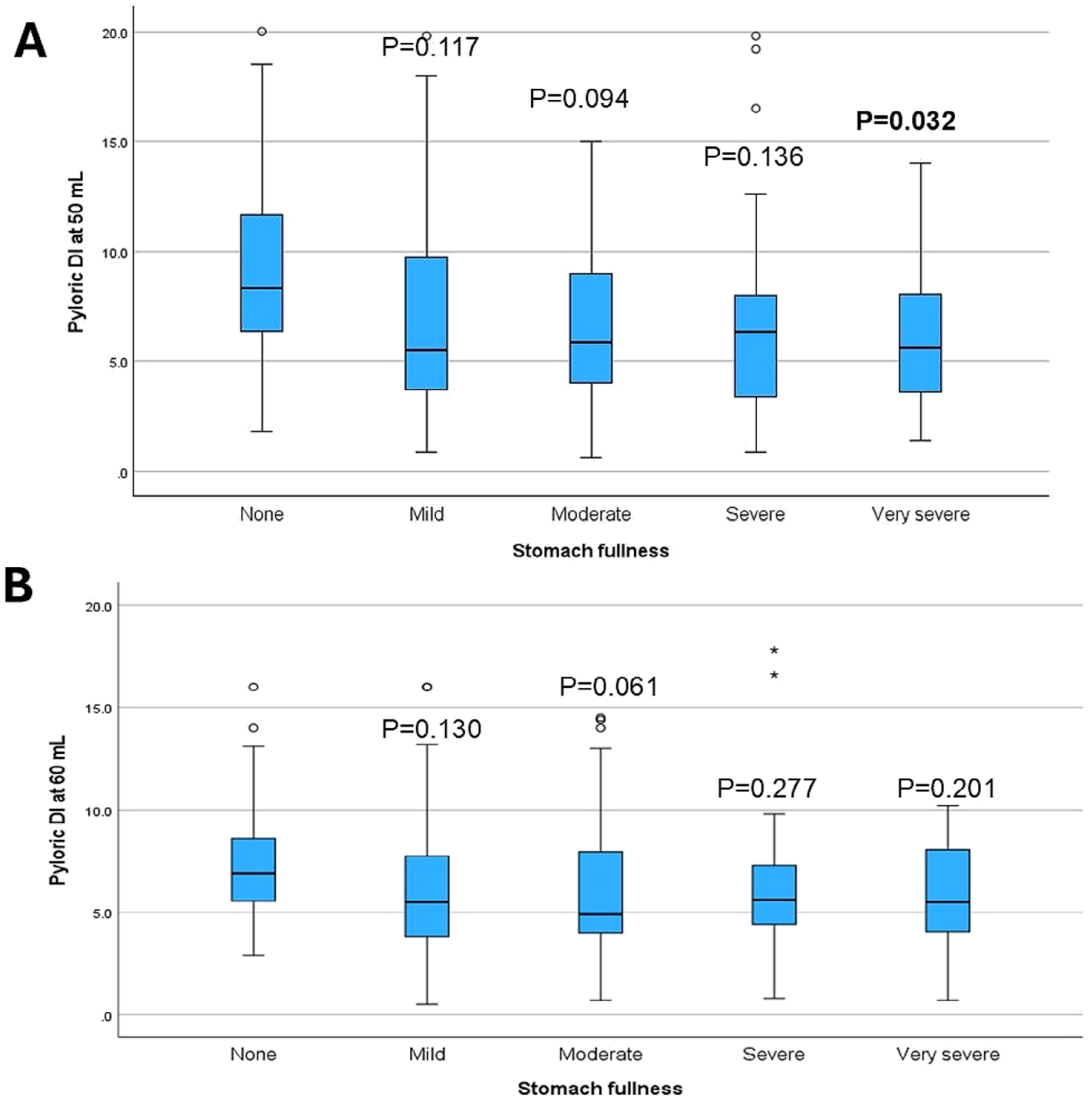
Comparison of median pyloric DI at 50 mL (A) and 60 mL (B) across different severities of stomach fullness. The *p*‐value represents the results of the non‐parametric independent sample test comparing DI in those without the symptom with those with different severity of the symptom.

For stomach fullness, DI at 40, 50, and 60 mL progressively decreased with increasing symptom severity. At 40 mL, DI was lower in those with mild (*p* = 0.016), moderate (*p* = 0.026), severe (*p* = 0.045) and very severe (*p* = 0.021) stomach fullness when compared with asymptomatic patients. However, these pairwise differences were not significant after applying the Bonferroni correction. At 50 mL too, we observed a similar trend with the DI progressively decreasing with increasing severity of stomach fullness. DI was smaller in those with mild (*p* = 0.012), moderate (*p* = 0.009), severe (*p* = 0.014) and very severe stomach fullness (*p* = 0.003) compared to those without this symptom. After applying Bonferroni correction, only the difference between patients with very severe fullness and no fullness was significant (5.6 [4.7] vs. 8.4 [5.3], *p* = 0.032; Figure 2A). A similar finding was seen at 60 mL, where DI was significantly smaller in those with mild (*p* = 0.013), moderate (*p* = 0.006), severe (*p* = 0.028) and very severe stomach fullness (*p* = 0.02) compared to those who were not experiencing this symptom. On applying the Bonferroni correction, however, only the difference between asymptomatic and those with moderate symptoms remained marginally significant (6.9 (3.4) vs. 4.9 (4.0), *p* = 0.061, Figure 2B). A weak inverse correlation was found between pyloric DI at 40, 50, and 60 mL and severity of fullness (*ρ* = −0.114, −0.162, and −0.119; *p* = 0.087, 0.014, and 0.077, respectively; Table 3).

**TABLE 3:** Receiver operating characteristic analysis of pyloric distensibility index for detection of delayed gastric emptying for 60–70 mL balloon volume.

Gastric retention after 4 h	Metric (DI at certain balloon volume)	Optimum cut‐off to diagnose delayed GE DI (mm^2^/mmHg)	Sensitivity	Specificity	Youden	AUC (95% C.I.)	*p*
> 10%	DI at 60 mL	≤ 8.90	91.0%	29.0%	0.196	0.61 (0.52–0.71)	0.047
	DI at 70 mL	≤ 6.25	89.0%	33%	0.222	0.61 (0.50–0.73)	0.056
> 20%	DI at 60 mL	≤ 8.9	94.7%	26.2%	0.209	0.61 (0.51–0.70)	0.029
	DI at 70 mL	≤ 6.25	92.5%	31%	0.229	0.62 (0.51–0.73)	0.038
> 30%	DI at 60 mL	≤ 8.0	90.2%	30%	0.202	0.62 (0.52–0.72)	0.022
	DI at 70 mL	≤ 4.80	82.8%	47%	0.290	0.66 (0.54–0.77)	0.009

For inability to finish a meal, overall differences in DI across the categories of severity were significant only at 70 mL (*p* = 0.028) but not at other volumes. Pairwise comparisons revealed that compared to those who did not report this symptom, DI was significantly higher in those with mild symptoms (5.1 (3.0) vs. 4.2 (2.0), *p* = 0.047) but this did not remain significant after applying the correction. DI was also smaller in those with moderate compared to mild symptoms (3.8 (2.2) vs. 5.1 (3.0), *p* = 0.001) and this remained significant even after applying the Bonferroni correction (*p* = 0.012).

A marginally significant difference in DI at 40 mL were seen across severity of loss of appetite (*p* = 0.076). Pairwise comparisons revealed that compared to those who did not report loss of appetite, DI was significantly smaller in those with moderate (4.9 (5.4) vs. 6.2 (4.8), *p* = 0.031) and severe loss of appetite (4.3 (4.6) vs. 6.2 (4.8), *p* = 0.037), but this did not remain significant after applying the correction.

No significant associations were observed between DI and severity of nausea, vomiting, retching, postprandial fullness, bloating, or abdominal distension.

#### 3.2.2 Area Under the Distensibility‐ Volume Curve (AUC)

**FIGURE 3:**
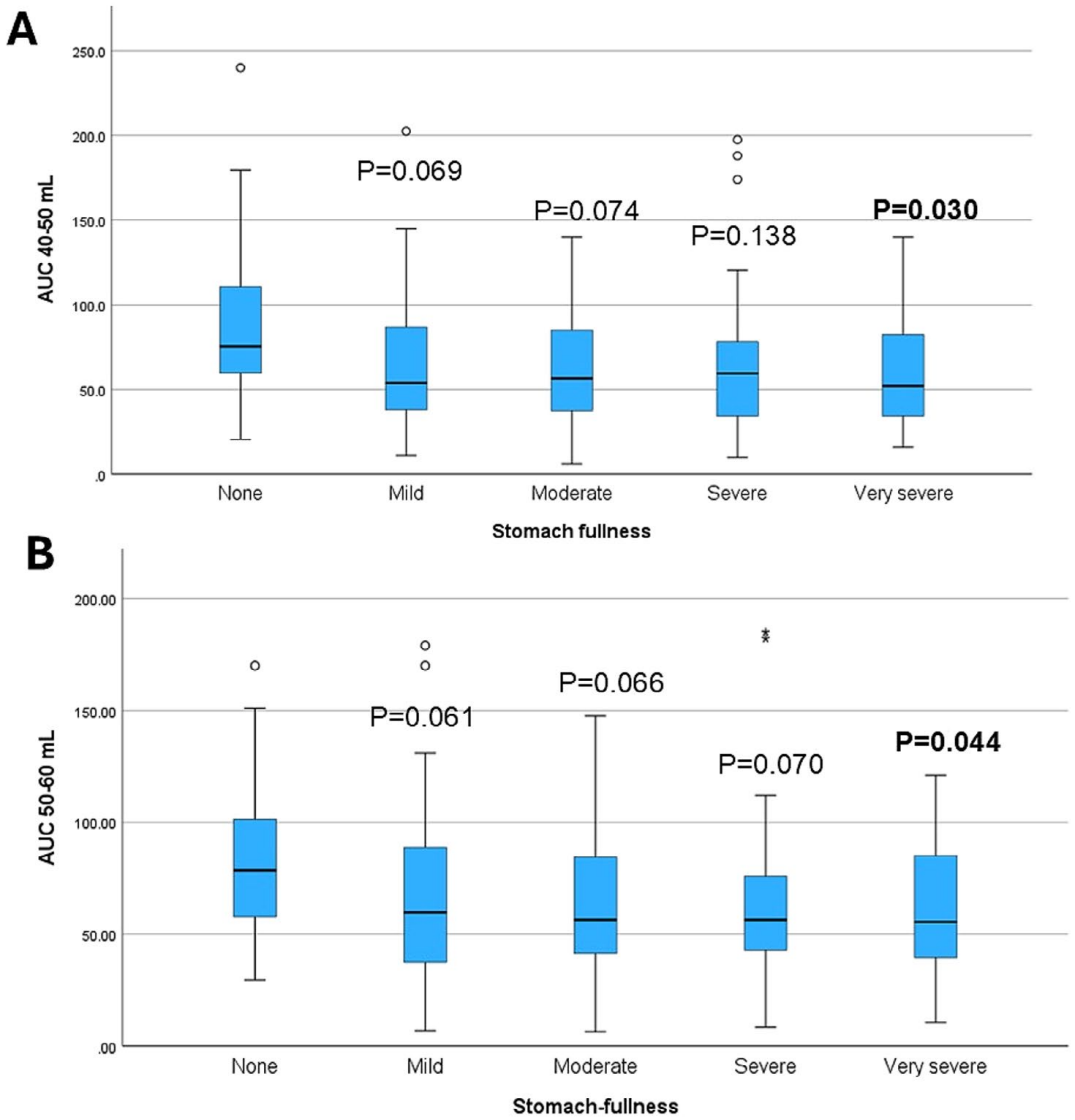
Comparison of median pyloric AUC at 40–50 mL (A) and 50–60 mL (B) across different severities of stomach fullness. The *p*‐value represents the results of a non‐parametric independent sample test comparing DI in those without the symptom with those with different severity of the symptom.

Next, we examined the differences in AUCs between patients with differing degrees of severity of gastroparesis symptoms. Stomach fullness was the only symptom where there was a difference in AUCs between groups (Table 2; Figure 3). There was a significant difference in the AUC_40–50mL_ across the different severities of the symptom of stomach fullness (p = 0.025). Pairwise comparison revealed that AUC was significantly smaller in those with mild (*p* = 0.007), moderate (*p* = 0.007), severe (*p* = 0.014), and very severe stomach fullness (*p* = 0.003) compared to those who were not experiencing this symptom. After Bonferroni adjustment, these differences remained significant for very severe stomach fullness (*p* = 0.030) and marginally significant for mild (*p* = 0.069) and moderate stomach fullness (*p* = 0.074). A similar pattern was seen for AUC_50–60mL_ where the AUC of patients with mild (*p* = 0.006), moderate (*p* = 0.007), severe (*p* = 0.007), and very severe (*p* = 0.004) symptoms were significantly smaller than that in patients not experiencing stomach fullness. After applying Bonferroni correction, the difference remained significant for those with very severe symptoms (*p* = 0.044) and marginally significant for mild (*p* = 0.061), moderate (*p* = 0.066), and severe stomach fullness (*p* = 0.07). AUC_60–70mL_ did not differ between varying severity of stomach fullness.

Pyloric AUC showed limited correlation with gastroparesis symptoms. AUCs between 40–50 mL and 50–60 mL showed a significant inverse correlation with the severity of stomach fullness (*ρ* = −0.157, *p* = 0.018 and *ρ* = −0.158, *p* = 0.019, respectively). No significant correlations were observed between pyloric AUC at other volumes and the severity of other gastroparesis symptoms.

### 3.3 ROC Analysis of Pyloric Distensibility for Identifying Delayed Gastric Emptying

Receiver operating characteristic (ROC) curve analyses were performed to evaluate the ability of pyloric DI to discriminate delayed gastric emptying defined by three different thresholds of gastric retention at 4 h. Pyloric DI at 40 mL and 50 mL showed poor discriminative ability to distinguish between those with normal vs. delayed gastric emptying at all three levels of gastric retention (10%, 20%, and 30%). The maximum Youden's index values were low across all distension volumes (< 0.10), indicating limited discriminatory ability (Table S4).

DI at 60 or 70 mL, however, had modest ability to discriminate between patients with normal and abnormal gastric retention (AUC of about 0.61–0.66). A DI of ≤ 8.9 mm^2^/mmHg at 60 mL and ≤ 6.25 mm^2^/mmHg at 70 mL identified those with a gastric retention of both > 10% and > 20% with a high sensitivity (91.0%–94.7% and 89.0%–92.5% respectively) but low specificity (26%–33%, Table 3).

At the highest retention threshold of 30%, DI at 70 mL demonstrated the strongest discriminatory performance, with an optimal cutoff of ≤ 4.8 mm^2^/mmHg, yielding a sensitivity of 82.8%, specificity of 47%, and the highest Youden's Index (0.290) across all analyses (AUC 0.66, 95% CI 0.54–0.77; *p* = 0.009). DI at 60 mL also discriminated this group, with an optimal cutoff of ≤ 8.0 mm^2^/mmHg (sensitivity 90.2%, specificity 30%; AUC 0.62, 95% CI 0.52–0.72; *p* = 0.022, Table 3).

### 3.4 Effect of Anesthesia on Pyloric Sphincter Distensibility

In a linear mixed‐effects model evaluating factors associated with pyloric distensibility index (DI), there was no significant overall main effect of anesthesia type on DI when averaged across all subjects and balloon distension volumes (*p* = 0.595). However, significant interaction effects were observed of anesthesia with balloon volume and gender (Table 4).

**TABLE 4:** Linear mixed‐effects model evaluating factors associated with pyloric distensibility index (DI) across increasing balloon distension volumes.

Variable	β (estimate)	95% CI	*p*
Anesthesia (General vs. Monitored)	−2.69	−5.58 to 0.20	0.068
Anesthesia × Gender (Female)	+3.49	1.07 to 5.91	0.005
Anesthesia × Volume (overall effect)	—	—	< 0.001
Gender (Female vs. Male)	−3.55	−5.74 to −1.35	0.002
BMI (per kg/m^2^)	−0.072	−0.120 to −0.024	0.003
Age (per year)	−0.003	−0.026 to 0.020	0.771

*Note:* Linear mixed‐effects model with pyloric distensibility index (DI, mm^2^/mmHg) as the dependent variable. Fixed effects included anesthesia type (general vs. monitored anesthesia care), gender, balloon distension volume (modeled as a continuous variable), and prespecified interaction terms (anesthesia × gender and anesthesia × volume). Models were adjusted for age and body mass index (BMI). A subject‐specific random intercept was included to account for repeated measurements within individuals, and a compound symmetry covariance structure was used for repeated measures across balloon volumes. Estimates represent the change in DI per unit increase in the predictor variable (per 1 mL for volume, per year for age, and per kg/m^2^ for BMI). Reference categories were monitored anesthesia care for anesthesia type and male sex for gender. Models were estimated using restricted maximum likelihood (REML) with Satterthwaite approximation for degrees of freedom. *p*‐values are derived from Type III tests of fixed effects.

**FIGURE 4:**
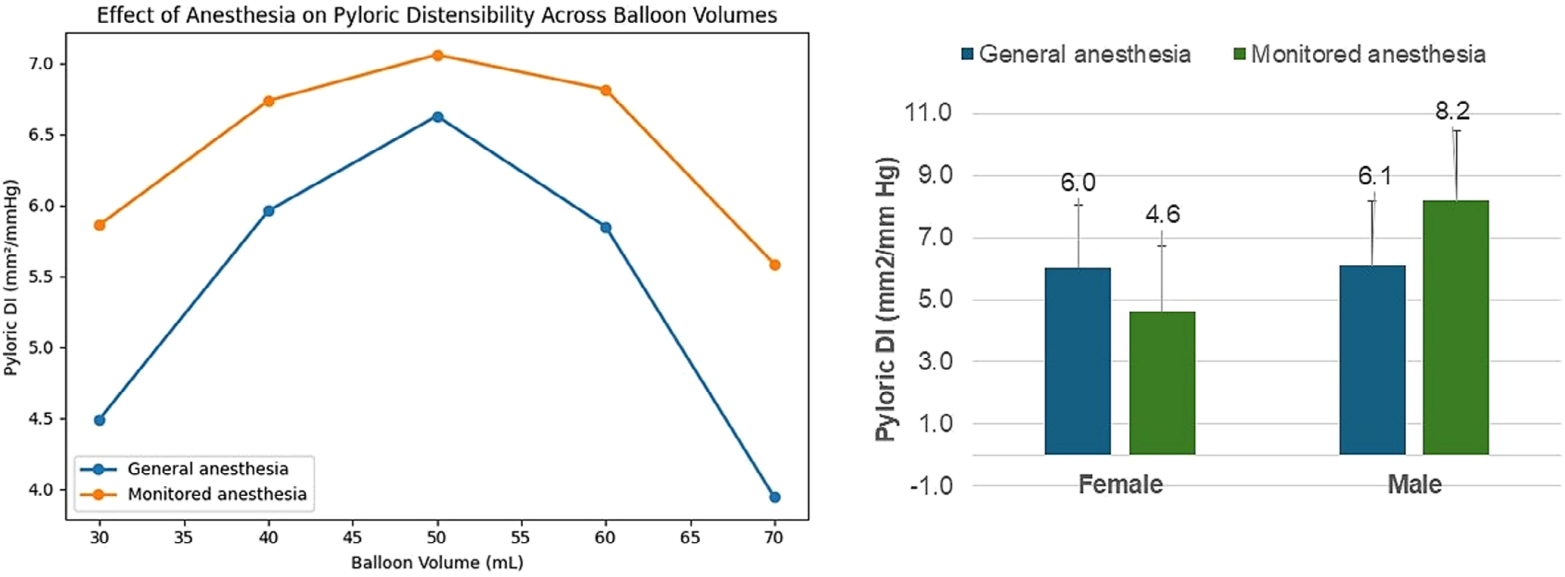
Effect of anesthesia type on pyloric distensibility index (DI) across balloon distension volumes. Adjusted estimated marginal means of DI are shown for general anesthesia and monitored anesthesia care across increasing balloon volumes (left) and sex (right) derived from the linear mixed‐effects model and adjusted for age and body mass index.

Specifically, a significant interaction was identified between type of anesthesia and balloon volume (*p* < 0.001), demonstrating that the effect of anesthesia on DI varied across increasing levels of balloon distension. Estimated marginal means showed that monitored anesthesia care was associated with consistently higher DI compared to general anesthesia across balloon volumes (Figure 4), with differences becoming more pronounced at higher distension levels. A significant interaction was also observed between anesthesia and gender (*p* = 0.005), indicating that the association between anesthesia type and pyloric distensibility differed between males and females.

Among covariates, female sex was independently associated with lower DI compared to males (β = −3.55, *p* = 0.002), and higher BMI was associated with lower DI (*β* = −0.072 per kg/m^2^, *p* = 0.003). Age, however, was not significantly associated with DI (*p* = 0.771).

## 4 Discussion

In this study, we evaluated the relationship between pyloric DI, AUC for the curve between pyloric DI and FLIP balloon volume with the severity of gastroparesis symptoms and the rate of gastric emptying. Our findings demonstrate that there is a statistically significant difference in pyloric DI and AUC, particularly at higher balloon volumes, between patients with and without gastroparesis. For most symptoms, there is no association between DI or AUC and GI symptoms except for stomach fullness, where there is a consistent decrease in DI and AUC at higher volumes. These results suggest that pyloric distensibility, particularly at higher volumes, may be a factor contributing to the delay in gastric emptying. Further, it highlights the limitation of pyloric distensibility assessment in explaining symptom severity.

We observed that pyloric DI measured at higher balloon volumes (60–70 mL) demonstrated stronger associations with delayed gastric emptying than measurements obtained at lower volumes. Non‐parametric comparisons showed statistically significant differences in DI at 60 and 70 mL across increasing thresholds of gastric retention at the 4‐h mark. The association was not observed at lower volumes. The association between pyloric DI and gastric emptying has been inconclusive in prior studies. One study in 54 gastroparesis patients found no association with the rate of gastric emptying [9]. However, that study used lower balloon volumes (20–50 mL) which may have under‐distended the pylorus. Another study using a catheter similar to the EF‐322 catheter that we used reported a negative correlation between pyloric DI at 40 mL and the T_1/2_ of gastric emptying in patients with gastroparesis [5]. A meta‐analysis published recently concluded that studies have not yet established a robust link between pyloric DI and the rate of gastric emptying [19]. Our study's uniqueness stems from examining across a wide range of balloon volumes which revealed significant associations that likely represent engagement of the pyloric ring and surrounding tissues by the EndoFLIP balloon at higher volumes, thereby uncovering pyloric outflow resistance which may not be evident at lower volumes. These findings suggest that pyloric dysfunction becomes more apparent under higher distension conditions, which may better approximate postprandial pyloric loading. These results will need to be confirmed by other studies.

The association between pyloric DI at higher FLIP balloon volumes and gastric emptying was further supported by the results of correlation and ROC analyses. In particular, DI measured at 60 and 70 mL showed weak negative correlations with gastric retention at 4 h, whereas correlations at earlier time points (1 and 2 h) were not statistically significant. The small magnitude of the correlations is consistent with the multifactorial pathophysiology of gastroparesis, in which delayed emptying reflects the combined effects of pyloric tone, antral hypomotility, impaired coordination, and vagal neuromodulation. DI at both 60 and 70 mL showed modest but statistically significant discriminatory ability for delayed gastric emptying using the ROC curve method, and their performance improved as the severity of gastric retention increased, particularly for DI at 70 mL. The highest diagnostic accuracy was observed for identifying > 30% retention using a DI threshold ≤ 4.8 at 70 mL, suggesting that pyloric DI especially at higher balloon volumes may serve as a screening or risk‐stratification marker for delayed gastric emptying. DI cut‐offs that identified delayed gastric emptying defined by more than 10% or 20% retention were similar (≤ 8.9 mm^2^/mmHg at 60 mL and ≤ 6.25 mm^2^/mmHg at 70 mL) suggest that these cut‐offs may be helpful to identify patients across a range of delays in gastric emptying. With recent studies suggesting that gastric retention more than 20% is associated with better clinical response to G‐POEM, Pyloric EndoFLIP may be helpful to identify patients who are more likely to improve with pyloric therapies such as dilation and POEM [20, 21]. Our study adds support to previous studies that have demonstrated that pyloric DI is inversely associated with presence of abnormal gastric retention [22].

Symptom associations and correlations with pyloric DI were however modest and limited to stomach fullness and inability to finish a regular sized meal. The inverse correlation between pyloric DI between 40 and 60 mL and severity of stomach fullness suggests that pyloric distensibility is a key contributor to the sensation of stomach fullness. The observation that pyloric DI showed a consistent association with gastric emptying but very selective association with GI symptoms suggests that symptoms may have other pathways (e.g., visceral sensation) or extra‐gastric origin (e.g., small bowel or colon) while gastric emptying is determined in large part by pyloric compliance. Our observations support previously noted associations between pyloric distensibility and gastroparesis symptoms [9], and supports the need for more research to elucidate the multifactorial pathophysiology involved in generating symptoms.

Given that measurement of pyloric DI without accounting for the volume may not provide us with the complete information about pyloric mechanics, we calculated a novel parameter, the AUC under the curve between the DI on the *y*‐axis and consecutive FLIP balloon volumes on the *x*‐axis. When pyloric function was summarized using these AUC measures, the pattern was similar to that with DI but the differences were more robust. In particular, AUCs at higher balloon volumes (50–60 mL and 60–70 mL) were significantly smaller in the group with abnormal gastric retention at 4 h across the three levels of severity (10%, 20%, and 30%). This suggests that AUC may be potentially a better metric than DI alone in distinguishing those with normal gastric emptying from those with gastroparesis. AUCs demonstrated a similar lack of association with symptoms with the exception of stomach fullness. AUCs in the mid‐volume ranges (40–50 mL and 50–60 mL) were significantly higher among patients who were free from the symptom of stomach fullness. Even mild symptoms were associated with a reduction in AUC at these volumes, suggesting that AUCs, gastric retention and stomach fullness are connected. A comparison of DI to AUC in predicting outcomes in patients with gastroparesis particularly after pyloric therapy remains to be investigated.

We observed that while the type of anesthesia (MAC or GA) does not have an overall effect on pyloric distensibility, there is a differential effect varying by both balloon distension volume and sex. Studies on the impact of anesthesia on pyloric DI are limited to just one—a multi‐center retrospective study comparing pyloric DI measurements using the EF‐325 catheter in 34 patients without general anesthesia with 91 patients with GA found that at 40 mL balloon volume, pyloric DI was smaller in those with GA (mean 8.7 vs. 11.6 in unsedated) but the difference was not statistically significant (*p* = 0.16) [23]. Thus, there is a lack of studies that have systematically examined the impact of anesthesia type on pyloric distensibility measurements. Our findings extend the existing literature by demonstrating a significant anesthesia × volume interaction, suggesting that general anesthesia may attenuate volume‐dependent increases in pyloric distensibility compared with monitored anesthesia care. In addition, the observed anesthesia × gender interaction raises the possibility of sex‐specific modulation of pyloric biomechanics under differing anesthetic states, a relationship that has not been previously described. Specifically, under general anesthesia, pyloric DI values were similar between males and females, suggesting a relative attenuation of sex‐related differences in pyloric biomechanics. In contrast, under monitored anesthesia care, a divergence emerged, with males demonstrating higher DI compared to females. This pattern suggests that general anesthesia may homogenize pyloric function across sexes, whereas monitored anesthesia may preserve or accentuate underlying physiologic differences. The mechanisms underlying this observation are unclear but may relate to differential effects of anesthetic agents on smooth muscle tone, autonomic regulation, or hormonal influences on gastrointestinal motility. Clinically, this finding raises the possibility that anesthesia type could differentially influence pyloric measurements in men and women, which may have implications for interpreting EndoFLIP‐derived metrics and establishing sex‐specific thresholds for intervention. However, these results should be interpreted with caution given the variability in subgroup estimates and wide confidence intervals, particularly within stratified analyses, and should be validated in larger, prospectively designed studies.

From a clinical perspective, our findings have several implications. First, pyloric EndoFLIP measurements obtained at higher balloon volumes showed an association with gastric emptying and may provide additional physiological insight beyond lower‐volume assessments. Second, the high sensitivity but limited specificity observed for most DI cutoffs on ROC analysis suggests that pyloric DI may be a useful phenotyping tool for patients with established, refractory symptoms. Third, the volume‐dependent and severity‐dependent nature of these associations supports a targeted approach to pylorus‐directed therapies. Patients with markedly reduced DI at higher balloon volumes and more severe gastric retention may represent a subgroup more likely to benefit from pyloric interventions such as G‐POEM, EsoFLIP, or pyloric botulinum toxin injection, although prospective validation is required. Notably, the pyloric distensibility index thresholds identified in our study are similar to those reported by Jacques et al., who demonstrated that reduced pyloric DI predicted symptomatic response to G‐POEM, further supporting the clinical relevance of EndoFLIP‐derived pyloric biomechanical metrics [24]. Specifically, the median DI at 50 mL was 6.9 mm^2^/mmHg and 7.2 mm^2^/mmHg for those with gastric retention of more than 20% or 40%, respectively, which was similar to the median DI at 50 mL for patients with Gastroparesis undergoing G‐POEM (8 mm^2^/mmHg).

A notable strength of this analysis is the substantially larger sample size compared with previously published pyloric EndoFLIP studies in gastroparesis, many of which have been limited by small cohorts and single‐volume measurements. The large sample size in the present study increases the likelihood that observed correlation estimates, though modest, mirror true physiological relationships rather than sampling variability. The lack of a strong association particularly with symptoms underscores the limitations of relying on single physiologic metrics to explain symptom burden and highlights the need for integrative phenotyping approaches that combine pyloric physiology with antral motility, sensory function, and central gut–brain interactions.

Despite this our study had some limitations. Symptom assessment relied on patient‐reported severity scales, which may be influenced by recall bias and central modulation. We did not subdivide gastroparesis by etiology, but previous research is mixed in terms of relationship between etiology of gastroparesis and FLIP parameters. We used the EF‐322 catheter because it allowed us to examine esophageal distensibility during the same procedure previous to that reported previously [13]. While many studies have used the EF‐325 catheter to investigate pyloric DI, our approach is supported by previous studies that have used the EF‐322 catheter to assess pyloric distensibility [13, 25]. In one study of over 180 patients, EndoFLIP assessment of the pyloric sphincter was investigated for its ability to identify pylorospasm in patients who presented for a variety of gastroesophageal symptoms [13]. The authors used data from either the EF‐325 and EF‐322 catheter to assess pyloric sphincter DI before and after G‐POEM procedure [25]. In a large multicenter study that examined role of EndoFLIP in predicting outcomes after G‐POEM, the authors used both the EF‐322 and the EF‐325 catheter for measurement of pyloric distensibility [25, 26]. They acknowledged that there is paucity of data comparing measurements using 8‐ and 16‐cm devices in the pylorus. They rationalized that both catheter type measurements were similar because DI and CSA are measured by a single electrode at the narrowest portion of the pylorus and the remaining electrodes are not used for these measurements. Recent studies suggest that there may be differences between measurements made using the two types of catheters (EF‐325 and EF‐322) [27]. There is need for more studies to establish the normative values using the EF‐322 catheter. Absolute DI values obtained via EF‐322 should be compared to EF‐325 reference data with caution due to the geometric differences between them. However, the use of the EF‐322 catheter can permit the assessment of pyloric and esophageal sphincter at the same time thus potentially contributing to a better understanding of gastroesophageal sphincter dysfunction. While pyloric diameter and cross‐sectional area (CSA) have been reported to be associated with gastroparesis symptoms, we focused on DI [9, 10]. We do not record CSA during our procedures. While diameter is recorded, we chose to focus our attention on DI for this manuscript. Future studies will examine the association of pyloric diameter with symptoms and gastric emptying. Including a gastric emptying study done within the last 10 years can potentially affect results as it could change significantly over a decade. Factors such as the development or progression of diabetes, changes in autonomic function, or new medications could certainly have altered a patient's GES results between the time of the scintigraphy and the EndoFLIP procedure. However, we wanted to increase the sensitivity of detecting gastroparesis in this “real‐world” scenario where many patients with gastroparesis have relatively stable (and often poorly controlled) symptoms over a long period of time. Future studies using a tighter interval between GES and EndoFLIP will clarify the association between pyloric dysfunction and gastric emptying better. In 2025, a cohort of French physicians comprising neurogastroenterologists and anesthesiologists issued a consensus statement describing the effect of anesthetics on digestive motility and proposed an anesthesia protocol for digestive motility assessment [28]. These recommendations underscore the importance of anesthetic standardization for future pyloric EndoFLIP studies. However, given the timing of this study and practical safety considerations during therapeutic endoscopy, complete adherence was not possible. Importantly, this limitation mirrors routine practice and supports the external validity of our findings. The possibility that pyloric distensibility is impacted by the dose of medications used during EndoFLIP procedure is an important one. In future studies, we will investigate the effect of doses of individual medications administered during anesthesia as well as other outpatient medications and medication groups. We used higher balloon volumes than previously reported for pyloric EndoFLIP. While balloon volumes up to 60–70 mL likely resulted in substantial pyloric stretching, these measurements were taken after visual confirmation of pyloric pressure and diameter stabilization. Consequently, DI and AUC reflect pyloric distensibility under stable distension conditions. Further, as concurrent antroduodenal manometry was not available, formal identification of phase 3 migratory motor complexes was not possible during EndoFLIP measurement. Measurements made after allowing for stabilzation of FLIP metrics are consistent with prior pyloric EndoFLIP studies [7, 10, 12]. All EndoFLIP procedures in our study were performed by a single endoscopist. While this ensured consistency of technique and also led to a new method to place the EndoFLIP catheter (the snare technique and gliding method), these findings need to be confirmed across other centers and providers.

## 5 Conclusions

In summary, pyloric distensibility measured using EndoFLIP demonstrates a modest association with delayed gastric emptying and select gastroparesis symptoms, particularly when assessed at higher balloon volumes and summarized using AUC metrics. These findings suggest that pyloric dysfunction is one component of gastroparesis pathophysiology. Further studies are needed to investigate the inclusion of EndoFLIP as part of a multi‐modal assessment alongside Gastric Emptying Scintigraphy (GES) and validated symptom scores to phenotype patients and inform targeted therapeutic strategies.

## Author Contributions

Subhankar Chakraborty: conceptualization, methodology, investigation, project administration, supervision, formal analysis, writing – original draft, writing – review and editing. Adam Spandorfer: investigation, data curation, formal analysis, writing – review and editing. Flavio Bonilla: investigation, data curation, formal analysis, writing – review and editing. Ladan Navari: investigation, data curation, formal analysis, writing – review and editing. All authors contributed substantially to the work, reviewed and approved the final manuscript, and agree to be accountable for all aspects of the study.

## Funding

The authors have nothing to report.

## Conflicts of Interest

The authors declare no conflicts of interest.

## Supporting information


**Table S1:** Demographics of the study population.

**Table S2:** Correlation of pyloric distensibility (DI) with symptoms of gastroparesis and percent gastric retention.

**Table S3:** Correlation of pyloric sphincter area under the curve (AUC) with symptoms of gastroparesis and percent gastric retention.

**Table S4:** Receiver operating characteristic analysis of pyloric distensibility index for detection of delayed gastric emptying for 40–50 mL balloon volume.

## Data Availability

Due to privacy and ethical concerns, data associated with this study are not available to share with other researchers.

## References

[1] M. Camilleri and K. M. Sanders , “Gastroparesis,” Gastroenterology 162 (2022): 68–87.e1. 34717924 10.1053/j.gastro.2021.10.028 PMC8678360

[2] A. Ardila‐Hani , M. Arabyan , A. Waxman , et al., “Severity of Dyspeptic Symptoms Correlates With Delayed and Early Variables of Gastric Emptying,” Digestive Diseases and Sciences 58 (2013): 478–487. 22918685 10.1007/s10620-012-2355-5

[3] H. K. Na , A. A. Li , A. Gottfried‐Blackmore , et al., “Pyloric Dysfunction: A Review of the Mechanisms, Diagnosis, and Treatment,” Gut Liver 19 (2025): 327–345. 40058793 10.5009/gnl240421 PMC12070220

[4] F. Wuestenberghs and G. Gourcerol , “Pyloric Distensibility in Health and Disease,” American Journal of Physiology. Gastrointestinal and Liver Physiology 321 (2021): G133–G138. 34160292 10.1152/ajpgi.00460.2020

[5] G. Gourcerol , F. Tissier , C. Melchior , et al., “Impaired Fasting Pyloric Compliance in Gastroparesis and the Therapeutic Response to Pyloric Dilatation,” Alimentary Pharmacology & Therapeutics 41 (2015): 360–367. 25523288 10.1111/apt.13053

[6] N. Jagtap , R. Kalapala , and D. N. Reddy , “Assessment of Pyloric Sphincter Physiology Using Functional Luminal Imaging Probe in Healthy Volunteers,” Journal of Neurogastroenterology and Motility 26 (2020): 391–396. 32606259 10.5056/jnm19200 PMC7329157

[7] C. Desprez , M. Chambaz , C. Melchior , et al., “Assessment of Pyloric Sphincter Distensibility and Pressure in Patients With Diabetic Gastroparesis,” Neurogastroenterology and Motility 33 (2021): e14064. 33314491 10.1111/nmo.14064

[8] T. Zheng , K. Vosoughi , I. Busciglio , L. Tebay , D. Burton , and M. Camilleri , “Fasting Pyloric Diameter and Distensibility by Functional Endoluminal Imaging Probe in Unsedated Healthy Volunteers,” Neurogastroenterology and Motility 34 (2022): e14386. 35468258 10.1111/nmo.14386 PMC9529766

[9] Z. Malik , A. Sankineni , and H. P. Parkman , “Assessing Pyloric Sphincter Pathophysiology Using EndoFLIP in Patients With Gastroparesis,” Neurogastroenterology and Motility 27 (2015): 524–531. 25712043 10.1111/nmo.12522

[10] M. Saadi , D. Yu , Z. Malik , H. P. Parkman , and R. Schey , “Pyloric Sphincter Characteristics Using EndoFLIP in Gastroparesis,” Revista de Gastroenterología de México (English Edition) 83 (2018): 375–384. 10.1016/j.rgmx.2018.02.013 29709494

[11] A. Jehangir , Z. Malik , R. V. Petrov , and H. P. Parkman , “EndoFLIP and Pyloric Dilation for Gastroparesis Symptoms Refractory to Pyloromyotomy/Pyloroplasty,” Digestive Diseases and Sciences 66 (2021): 2682–2690. 32749636 10.1007/s10620-020-06510-0

[12] C. Desprez , C. Melchior , F. Wuestenberghs , et al., “Pyloric Distensibility Measurement Predicts Symptomatic Response to Intrapyloric Botulinum Toxin Injection,” Gastrointestinal Endoscopy 90 (2019): 754–760.e1. 31028783 10.1016/j.gie.2019.04.228

[13] I. Levenfus , A. Bianca , J. Hente , et al., “Subjective Assessment of the Pyloric Sphincter During Endoscopy and Its Correlation With FLIP Panometry,” Digestive Diseases and Sciences 70 (2025): 3432–3442. 40579596 10.1007/s10620-025-09127-3 PMC12531320

[14] C. Varghese , A. Lim , C. Daker , et al., “Predictors of Outcomes After Gastric Peroral Endoscopic Myotomy for Refractory Gastroparesis: A Systematic Review,” American Journal of Gastroenterology 120 (2024): 1275–1284. 39733275 10.14309/ajg.0000000000003213

[15] J. L. Carlin , V. R. Lieberman , A. Dahal , et al., “Efficacy and Safety of Tradipitant in Patients With Diabetic and Idiopathic Gastroparesis in a Randomized, Placebo‐Controlled Trial,” Gastroenterology 160 (2021): 76–87.e4. 32693185 10.1053/j.gastro.2020.07.029

[16] A. S. Msdi , A. F. Ravari , J. C. Abdul‐Mutakabbir , and K. K. Tan , “Are All Pharmacokinetic Equations Created Equal? A Comparative Analysis of Trapezoidal and Non‐Trapezoidal Methods for Estimating Day 1 Area Under the Curve in Adult Hospitalized Patients With *Staphylococcus aureus* Bacteremia,” Infectious Diseases and Therapy 14 (2025): 615–626. 39962022 10.1007/s40121-025-01115-4 PMC11933637

[17] A. D. Dunvald , D. B. Iversen , A. L. O. Svendsen , et al., “Tutorial: Statistical Analysis and Reporting of Clinical Pharmacokinetic Studies,” Clinical and Translational Science 15 (2022): 1856–1866. 35570335 10.1111/cts.13305 PMC9372427

[18] D. A. Revicki , A. M. Rentz , D. Dubois , et al., “Gastroparesis Cardinal Symptom Index (GCSI): Development and Validation of a Patient Reported Assessment of Severity of Gastroparesis Symptoms,” Quality of Life Research 13 (2004): 833–844. 15129893 10.1023/B:QURE.0000021689.86296.e4

[19] A. Barchi , M. Gupta , N. Warringa , et al., “Endoluminal Functional Lumen Imaging Probe in the Functional Assessment of Pyloric Sphincter in Gastroparesis: A Systematic Review With Meta‐Analysis of Normative Values,” Neurogastroenterology and Motility 37 (2025): e70187. 41133405 10.1111/nmo.70187

[20] J. Martinek , R. Hustak , J. Mares , et al., “Endoscopic Pyloromyotomy for the Treatment of Severe and Refractory Gastroparesis: A Pilot, Randomised, Sham‐Controlled Trial,” Gut 71 (2022): 2170–2178. 35470243 10.1136/gutjnl-2022-326904 PMC9554080

[21] P. Mekaroonkamol , V. Patel , R. Shah , et al., “Association Between Duration or Etiology of Gastroparesis and Clinical Response After Gastric Per‐Oral Endoscopic Pyloromyotomy,” Gastrointestinal Endoscopy 89 (2019): 969–976. 30653937 10.1016/j.gie.2018.12.023

[22] W. J. Snape , M. S. Lin , N. Agarwal , and R. E. Shaw , “Evaluation of the Pylorus With Concurrent Intraluminal Pressure and EndoFLIP in Patients With Nausea and Vomiting,” Neurogastroenterology and Motility 28 (2016): 758–764. 26813266 10.1111/nmo.12772

[23] C. Desprez , J. Jacques , T. Clavier , T. Wallenhorst , A. M. Leroi , and G. Gourcerol , “Impact of Anesthetics on Pyloric Characteristics Measured Using the EndoFLIP System in Patients With Gastroparesis,” Neurogastroenterology and Motility 35 (2023): e14651. 37496304 10.1111/nmo.14651

[24] J. Jacques , L. Pagnon , F. Hure , et al., “Peroral Endoscopic Pyloromyotomy Is Efficacious and Safe for Refractory Gastroparesis: Prospective Trial With Assessment of Pyloric Function,” Endoscopy 51 (2019): 40–49. 29895073 10.1055/a-0628-6639

[25] K. Vosoughi , Y. Ichkhanian , J. Jacques , et al., “Role of Endoscopic Functional Luminal Imaging Probe in Predicting the Outcome of Gastric Peroral Endoscopic Pyloromyotomy (With Video),” Gastrointestinal Endoscopy 91 (2020): 1289–1299. 32035074 10.1016/j.gie.2020.01.044

[26] L. S. Watts , J. R. Baker , A. A. Lee , et al., “Impact of Gastric Per‐Oral Endoscopic Myotomy on Static and Dynamic Pyloric Function in Gastroparesis Patients,” Neurogastroenterology and Motility 32 (2020): e13892. 32542920 10.1111/nmo.13892

[27] I. Hirano , J. E. Pandolfino , and G. E. Boeckxstaens , “Functional Lumen Imaging Probe for the Management of Esophageal Disorders: Expert Review From the Clinical Practice Updates Committee of the AGA Institute,” Clinical Gastroenterology and Hepatology 15 (2017): 325–334. 28212976 10.1016/j.cgh.2016.10.022 PMC5757507

[28] D. Renard , T. Clavier , O. Abou‐Arab , et al., “Consensus on the Management of Anesthetic Agents During Digestive Motility Measurements and Proposal of a Standardized Protocol for Anesthesia (French Neuro Gastroenterology Group GFNG and Committee of Anesthetic French Experts),” Neurogastroenterology and Motility 38 (2025): e70098. 40528713 10.1111/nmo.70098 PMC13121879

