# Supplementary material for: EndoFLIP Guided Assessment of Pyloric Distensibility Identifies Associations With Delayed Gastric Emptying and Symptoms of Gastroparesis
Source: Neurogastroenterol Motil. 2026 Jul 27;38(7):e70405. doi: 10.1111/nmo.70405 (PMC13402978; doi:10.1111/nmo.70405)
Supplement: Supplementary file 1 — Table S1: Demographics of the study population. [file NMO-38-e70405-s004.docx]

**Supplementary Table 1. Demographics of the study population**

|  | Median (IQR) |  |
| --- | --- | --- |
| Clinical characteristics | Included (N=229) | Excluded (N=92) |
| Completed symptom questionnaire? | Yes | No |
| Age (years) | 50.0 (20) | 52.0 (26) |
| Females (N, %) | 183 (79.9%) | 71 (77.2%) |
| White Race | 204 (89.1%) | 68 (73.9%) |
| Diabetic status |  |  |
| Diabetic | 54 (23.6%) |  |
| Non-diabetic | 173 (75.5%) |  |
| Gastric Emptying results (N=132) |  |  |
| Normal | 64 (48.5%) |  |
| Delayed | 68 (51.5%) |  |
| Anesthesia type |  |  |
| Monitored anesthesia | 33 (14.3%) |  |
| General anesthesia | 198 (85.7%) |  |
