# Supplementary material for: EndoFLIP Guided Assessment of Pyloric Distensibility Identifies Associations With Delayed Gastric Emptying and Symptoms of Gastroparesis
Source: Neurogastroenterol Motil. 2026 Jul 27;38(7):e70405. doi: 10.1111/nmo.70405 (PMC13402978; doi:10.1111/nmo.70405)
Supplement: Supplementary file 2 — Table S2: Correlation of pyloric distensibility (DI) with symptoms of gastroparesis and percent gastric retention. [file NMO-38-e70405-s001.docx]

**Supplementary Table 2. Correlation of pyloric distensibility (DI) with symptoms of gastroparesis and percent gastric retention**

|  |  | **DI 40 mL** | **DI 50 mL** | **DI 60 mL** | **DI 70 mL** |
| --- | --- | --- | --- | --- | --- |
| Gastric retention |  |  |  |  |  |
| After 1 hr | ρ | -0.010 | -0.077 | -0.126 | -0.150 |
|  | p-value | 0.896 | 0.334 | 0.128 | 0.142 |
| After 2 hrs | ρ | -0.100 | -0.069 | -0.102 | -0.065 |
|  | p-value | 0.215 | 0.391 | 0.218 | 0.531 |
| After 4 hrs | ρ | **-0.147** | **-0.139** | **-0.239** | **-0.260** |
|  | p-value | **0.078** | **0.093** | **0.005** | **0.011** |
| Nausea | ρ | 0.030 | -0.025 | -0.008 | 0.083 |
|  | p-value | 0.654 | 0.707 | 0.911 | 0.322 |
| Retching | ρ | 0.084 | -0.009 | -0.015 | 0.023 |
|  | p-value | 0.209 | 0.890 | 0.793 | 0.781 |
| Vomiting | ρ | 0.082 | 0.033 | 0.046 | 0.066 |
|  | p-value | 0.222 | 0.619 | 0.499 | 0.431 |
| Stomach fullness | ρ | -0.114 | **-0.162** | -0.119 | -0.014 |
|  | p-value | 0.087 | **0.014** | 0.077 | 0.863 |
| Inability to finish a meal | ρ | 0.035 | 0.004 | -0.023 | -0.057 |
|  | p-value | 0.604 | 0.950 | 0.730 | 0.489 |
| Excessive post prandial fullness | ρ | -0.048 | -0.114 | -0.060 | 0.017 |
|  | p-value | 0.479 | 0.087 | 0.376 | 0.839 |
| Loss of appetite | ρ | -0.095 | -0.101 | -0.064 | -0.016 |
|  | p-value | 0.153 | 0.128 | 0.343 | 0.843 |
| Bloating | ρ | -0.003 | -0.032 | -0.029 | -0.039 |
|  | p-value | 0.966 | 0.636 | 0.671 | 0.642 |
| Abdominal distension | ρ | -0.054 | -0.081 | -0.034 | -0.057 |
|  | p-value | 0.421 | 0.221 | 0.611 | 0.490 |

*Footnote:* Table represents the results of Spearman’s correlation coefficient (ρ) and the p-value of the correlation
