# Supplementary material for: EndoFLIP Guided Assessment of Pyloric Distensibility Identifies Associations With Delayed Gastric Emptying and Symptoms of Gastroparesis
Source: Neurogastroenterol Motil. 2026 Jul 27;38(7):e70405. doi: 10.1111/nmo.70405 (PMC13402978; doi:10.1111/nmo.70405)
Supplement: Supplementary file 3 — Table S3: Correlation of pyloric sphincter area under the curve (AUC) with symptoms of gastroparesis and percent gastric retention. [file NMO-38-e70405-s003.docx]

**Supplementary Table 3. Correlation of pyloric sphincter area under the curve (AUC) with symptoms of gastroparesis and percent gastric retention**

|  |  | AUC 40-50 mL | AUC 50-60 mL | AUC 60-70 mL |
| --- | --- | --- | --- | --- |
| Gastric retention |  |  |  |  |
| After 1 hr | ρ | -0.045 | -0.098 | -0.159 |
|  | p-value | 0.575 | 0.236 | 0.119 |
| After 2 hrs | ρ | -0.084 | -0.088 | -0.090 |
|  | p-value | 0.298 | 0.291 | 0.383 |
| After 4 hrs | ρ | -0.140 | **-0.206** | **-0.264** |
|  | p-value | 0.094 | **0.015** | **0.010** |
| Nausea | ρ | -0.007 | -0.012 | 0.093 |
|  | p-value | 0.916 | 0.858 | 0.265 |
| Retching | ρ | 0.028 | -0.006 | 0.070 |
|  | p-value | 0.678 | 0.934 | 0.398 |
| Vomiting | ρ | 0.052 | 0.038 | 0.108 |
|  | p-value | 0.444 | 0.577 | 0.198 |
| Stomach fullness | ρ | **-0.157** | **-0.158** | -0.049 |
|  | p-value | **0.018** | **0.019** | 0.556 |
| Inability to finish a meal | ρ | 0.013 | -0.011 | -0.038 |
|  | p-value | 0.848 | 0.869 | 0.647 |
| Excessive post prandial fullness | ρ | -0.093 | -0.094 | -0.006 |
|  | p-value | 0.169 | 0.165 | 0.940 |
| Loss of appetite | ρ | -0.117 | -0.075 | -0.005 |
|  | p-value | 0.079 | 0.270 | 0.951 |
| Bloating | ρ | -0.029 | -0.042 | -0.009 |
|  | p-value | 0.667 | 0.535 | 0.910 |
| Abdominal distension | ρ | -0.081 | -0.065 | -0.045 |
|  | p-value | 0.226 | 0.333 | 0.589 |

*Footnote:* Table represents the results of Spearman’s correlation coefficient (ρ) and the p-value of the correlation
