# Supplementary material for: EndoFLIP Guided Assessment of Pyloric Distensibility Identifies Associations With Delayed Gastric Emptying and Symptoms of Gastroparesis
Source: Neurogastroenterol Motil. 2026 Jul 27;38(7):e70405. doi: 10.1111/nmo.70405 (PMC13402978; doi:10.1111/nmo.70405)
Supplement: Supplementary file 4 — Table S4: Receiver operating characteristic analysis of pyloric distensibility index for detection of delayed gastric emptying for 40–50 mL balloon volume. [file NMO-38-e70405-s002.docx]

**Supplementary Table 4: Receiver operating characteristic analysis of pyloric distensibility index for detection of delayed gastric emptying for 40-50 mL balloon volume**

| Gastric retention after 4 hrs. | Metric (DI at certain balloon volume) | AUC | 95% C.I. | p-value |
| --- | --- | --- | --- | --- |
| > 10% | 40 mL | 0.475 | 0.382-0.568 | 0.598 |
|  | 50 mL | 0.436 | 0.344-0.528 | 0.175 |
|  |  |  |  |  |
| > 20% | 40 mL | 0.412 | 0.312-0.512 | 0.084 |
|  | 50 mL | 0.432 | 0.330-0.533 | 0.187 |
|  |  |  |  |  |
| > 30% | 40 mL | 0.422 | 0.309-0.534 | 0.173 |
|  | 50 mL | 0.418 | 0.309-0.527 | 0.138 |
